# Paroxetine-induced galactorrhea

**DOI:** 10.1192/j.eurpsy.2022.2265

**Published:** 2022-09-01

**Authors:** S. Petrykiv, M. Arts, L. De Jonge

**Affiliations:** 1 GGZWNB, Psychiatry, Halsteren, Netherlands; 2 GGZWNB, Psychiatry, Bergen op Zoom, Netherlands; 3 Leonardo Scientific Research Institute, Neuropsychiatry, Bergen op Zoom, Netherlands

**Keywords:** Galactorrhea, Paroxetine, Case discussion, Literature review

## Abstract

**Introduction:**

Antidepressant-induced galactorrhea and increases in prolactin levels have been sporadically reported among SSRI-related side effects.

**Objectives:**

Current rapport presents a case of 39 y.o. female who developed several adverse effects on paroxetine - including galactorrhoea - which improved on discontinuation of the drug.

**Methods:**

Case discussion of 39-year old woman who was treated with paroxetine for her panic disorder and developed galactorrhoea with hyperprolactinemia that resolved upon discontinuation of the drug. Additionally, authors performed the literature search using PubMed and Embase to review the similar cases and used PDSP Database to assess the latest pharmacodynamic (PD) properties of paroxetine and other SSRI’s.

**Results:**

Literature review (1966–2020) revealed 24 prior published case reports of SSRI-induced galactorrhea in users of paroxetine (n=4), escitalopram (n=4), sertraline (n=2), citalopram (n=2), fluoxetine (n=3), fluvoxamine (n=2) and other non-assessable reports (n=7). Elevated prolactin levels were mostly observed with paroxetine and escitalopram and rarely with fluoxetine, fluvoxamine and sertraline. PD-assessment showed the highest binding affinity of paroxetine and escitalopram to SERT (kPi = 0.07-0.2 and 0.8-1.1 nmol/L respectively) compared to other SSRI’s, in absence of other relevant PD-properties

**Conclusions:**

Increasing body of evidence shows that galactorrhea does occur among paroxetine female users. Pharmacodynamic mechanism of action is poorly understood but given the modern insights in relationship in serotonin and dopamine circuits, we suggest that strong SERT inhibitory properties of paroxetine might lead to a tonic suppressive influence on dopamine neurotransmission. This physiological link may explain an increase in prolactin levels through dopamine depletion in the tuberoinfundibular pathway.

**Disclosure:**

No significant relationships.

